# The effects of combined balance and plyometric training on change-of-direction and dynamic balance: A meta-analysis

**DOI:** 10.1371/journal.pone.0346232

**Published:** 2026-03-31

**Authors:** Guang Feng, Ruobing Chen, Chonghui Wu, Yongfeng Liu

**Affiliations:** 1 School of Sports Training, Chengdu Sport University, Chengdu, China; 2 School of Foreign Languages, Xihua University, Chengdu, China; Lorestan University, IRAN, ISLAMIC REPUBLIC OF

## Abstract

**Background:**

Preceding studies have demonstrated that a combination of balance and plyometric training can enhance change-of-direction and dynamic balance. However, to date, a paucity of meta-analyses has precluded the provision of a comprehensive summary of the extant data.

**Objective:**

The meta-analysis aims to examine the effects of combined balance and plyometric training on change-of-direction and dynamic balance compared to active controls.

**Method:**

The present meta-analysis was conducted in strict adherence to the PRISMA 2020 guidelines. A systematic search was conducted in five electronic databases: PubMed, Web of Science, Scopus, Embase, EBSCOhost. The present study incorporated studies published from inception until June 2025. Eligibility was assessed using the PICOS method. The quality of studies was assessed using the Cochrane risk of bias assessment tool. For the meta-analysis, the random-effects model was utilised, and the Hedges’ g effect size (ES) and its 95% confidence interval (95% CI) were reported. Subgroup analyses were conducted (age, gender, training frequency, duration).

**Results:**

A total of 10 studies with 270 participants were included. Compared with the control group, combined balance and plyometric training significantly improved change-of-direction (ES = −0.77, *I*^2^ = 65.6%, *P* < 0.05), Y-Balance (ES = 1.32, *I*² = 67.5%, *P* < 0.05), dynamic postural stability index (ES = −1.27, I² = 0.0%, *P* < 0.05), and center of pressure (ES = −1.25, *I*² = 58.8%, *P* < 0.05). Subgroup analysis showed that age, gender, training frequency, and duration had no effect on the training effect of change-of-direction.

**Conclusions:**

Combined balance and plyometric training can significantly improve change-of-direction and dynamic balance, and existing evidence shows that the training effects of change-of-direction are not affected by age, gender, training frequency, or duration.

## 1. Introduction

In sports, optimizing athletic performance and preventing sports injuries are the core of physical fitness training. Among these, change-of-direction and dynamic balance abilities are key indicators for measuring lower limb coordination, neuromuscular control levels, and athletic performance, and are of great significance in sports such as basketball, soccer, rugby, and badminton [1,2]. Good change-of-direction ability helps athletes quickly adapt to various sudden changes and quickly make adjustments, while good dynamic balance ability is the basis for maintaining physical stability and preventing non-contact injuries [3].

In recent years, researchers have proposed a variety of training methods to improve change-of-direction and dynamic balance abilities, among which plyometric training and balance training have been widely used. Plyometric training promotes neuromuscular reaction speed and muscle strength output through a stretch-shortening cycle mechanism [4–6], while balance training improves the body's ability to regulate posture in unstable environments by improving proprioception and small muscle group control [7]. Although the extensive research that has already been conducted on the individual effects of balance and plyometric training, recent studies have increasingly focused on the potential synergistic benefits of combining these two types of training. Preliminary evidence shows that combined balance and plyometric training has greater advantages in improving change-of-direction and dynamic balance abilities [8,9].

However, to date, a paucity of meta-analyses has precluded the provision of a comprehensive summary of the extant data. Compared to individual experiments, meta-analyses can integrate the results of multiple studies and provide greater accuracy [10]. Therefore, we plan to conduct a meta-analysis focusing on the effects of combined balance and plyometric training on change-of-direction and dynamic balance abilities, and analyze whether the training effect is influenced by participant characteristics and training programs.

## 2. Methods

This meta-analysis was performed in adherence to the 2020 PRISMA guidelines [11]. Furthermore, it is evident that the protocol for the present meta-analysis has been prospectively registered with the International Prospective Register of Systematic Reviews (PROSPERO), assigned the unique registration identifier CRD420251085579.

### 2.1. Literature search strategy

This meta-analysis employed Boolean operators “AND” and “OR” to conduct a comprehensive search across five databases: PubMed, Web of Science, Scopus, Embase, EBSCOhost. The S1 Table 1 provides detailed search strategies for each database. Search terms comprised “plyometric,” “force,” “velocity,” “stretch,” “shortening cycle,” “balance,” “instability,” “training” and “posture control.” Additionally, to make sure we didn't miss any relevant studies, we checked the reference lists of the included articles and reviews. All randomized controlled trials published before June 2025 were included.

### 2.2. Inclusion and exclusion criteria

The following criteria were used to include studies in this meta-analysis, which was based on the PICOS principle: (1) healthy participants, with no restrictions on age, gender, or exercise level; (2) interventions using combined balance and plyometric training; (3) control groups using separate plyometric training, separate balance training, or routine training; (4) At least one outcome measure related to change-of-direction or dynamic balance; (5) A randomised controlled trial was the study design. Research was ruled out if it (1) was a review article, duplicate publication, conference summary, letter to the editor, special circular, case report, or acute study;(2) was a publication for which the complete text or original data were not accessible.

### 2.3. Selection process

The literature screening process is as follows: First, researcher GF used literature management software (EndNote 20) to remove duplicate records and assigned the remaining articles to two independent researchers (GF and RBC). Second, GF and RBC independently assessed the titles and abstracts of each article based on predefined exclusion and inclusion criteria. Finally, all articles that passed the initial screening were reviewed in full by GF and RBC to determine their final inclusion. It is important to note that throughout the screening process, should there be any disagreement between the two researchers (GF and RBC) regarding the eligibility of an article for inclusion, a third independent researcher (YFL) would conduct a comprehensive review of the disputed article. The purpose of this review would be to determine whether the article should proceed to the next stage of review.

### 2.4. Data extraction

Two researchers independently extracted all research data. In cases of disagreement between the two researchers, the issue was resolved through consensus or the opinion of a third researcher. We primarily extracted the following information: publication year, first author, participant characteristics (including the number of participants in the experimental and control groups, gender, and age), intervention protocol information (training duration, training frequency, whether supervised, etc.), study design information (whether blinded, etc.), and the mean, standard deviation, and sample size for each outcome measure before and after the intervention. If any information was missing, the researchers got in touch with the authors of the included studies by email to ask for the missing information, where required.

### 2.5. Risk of bias and quality of methods assessment

The risk of bias in the included studies was assessed independently by two researchers using the Cochrane Risk of Bias Tool. This tool comprises seven distinct assessment items: a) randomization method, b) allocation concealment, c) blinding of participants and personnel, d) blinding of outcome assessment, e) completeness of outcome data, f) selective reporting of study results, and g) other sources of bias [12]. The overall risk of bias assessment did not include this assessment item because it is difficult to blind participants in exercise interventions. Instead, blinding of outcome assessment by operators was considered a quality standard. We then gave each study an overall risk of bias score: 5 points or higher was considered low bias risk; 3–4 points was considered moderate bias risk; and less than 3 points was considered high bias risk.

### 2.6. Data synthesis and statistical analyses

The Cochrane Handbook stipulates that a meta-analysis may be conducted with as few as two studies. However, given the typically small sample sizes in sports science research, we only perform meta-analyses when at least three studies are available [13]. The alterations from baseline to post-intervention in both the experimental and control groups were aggregated to calculate the effect sizes. The standard deviation (SD) of the change was calculated using the formula provided in the Cochrane Handbook for Systematic Reviews of Interventions (version 6.3) [14]. The formula is as follows:


$${\text{SD}}_{\text{change}}=\sqrt{\overset{2}{\underset{\text{baseline}}{\text{SD}}}+\overset{2}{\underset{\text{final}}{\text{SD}}}-(2\times Corr\times {\text{SD}}_{\text{baseline}}\times {\text{SD}}_{\text{final}})}$$

Stata 17 software was used to conduct a meta-analysis. Due to differences in the measurement methods and units of the outcome measures, the standardized mean difference (SMD) and 95% confidence interval (CI) were used for statistical pooling. The effect size was expressed using ES (Hedge's g), and the effect sizes were classified according to the following criteria: Very small (<0.2), small (0.2–0.5), moderate (>0.5–0.8), and large (>0.8) [15]. A random-effects model was uniformly applied for the meta-analysis [16]. The *I*² value was used to assess heterogeneity, with *I*² < 25% indicating no heterogeneity, 25%−50% indicating mild heterogeneity, 50%−75% indicating moderate heterogeneity, and >75% indicating severe heterogeneity [17]. When *I*² > 50%, sensitivity analysis was conducted using a leave-one-out method to assess heterogeneity. Egger's asymmetry test were used to assess publication bias [18,19]. Additionally, subgroup analyses were conducted to investigate the sources of heterogeneity and explore the effects of variables such as age, gender, training frequency and duration on training outcomes.

## 3. Results

### 3.1. Literature selection

**Fig 1:**
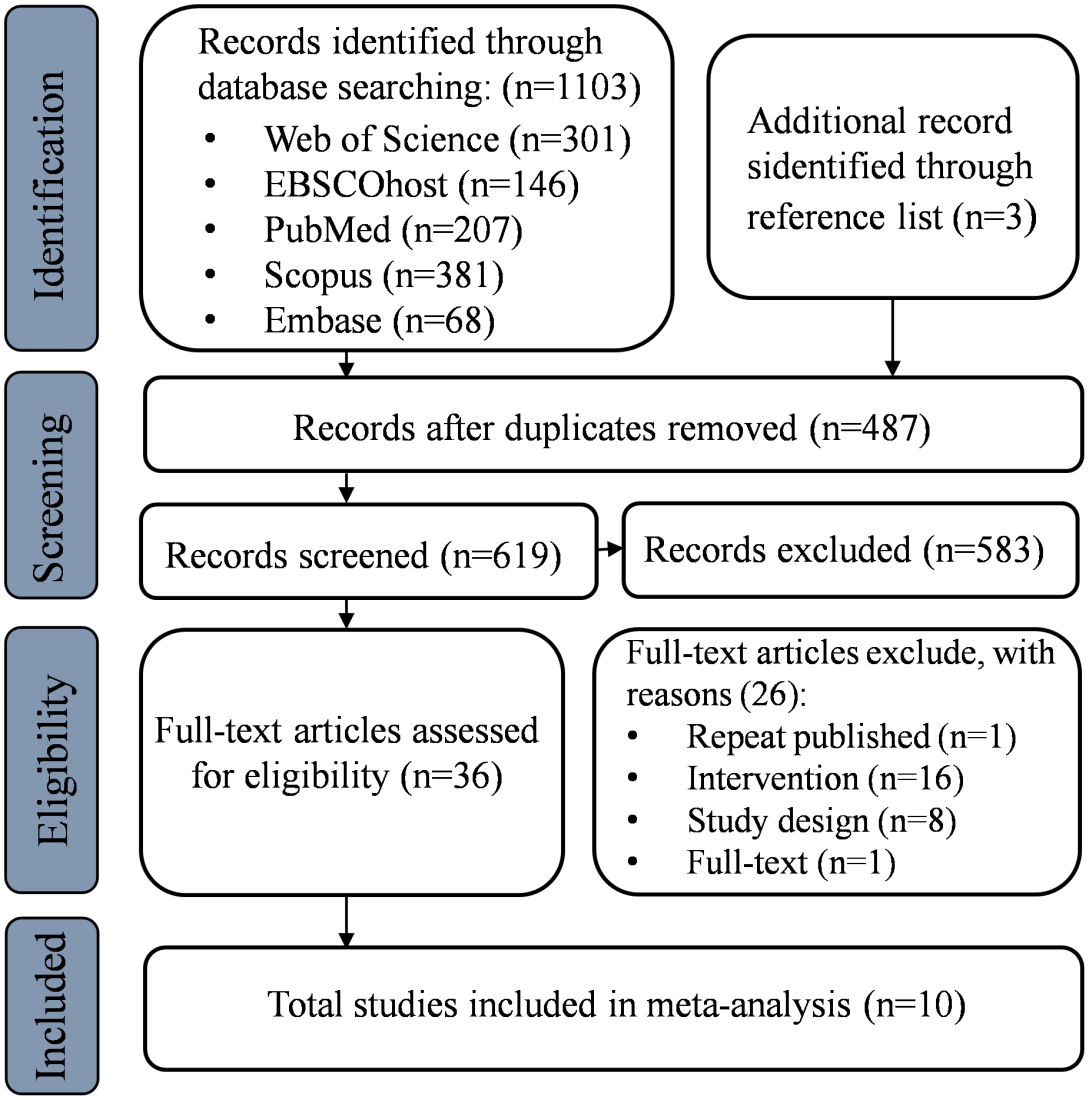
Preferred Reporting Items for Systematic Reviews and Meta-Analyses (PRISMA) flowchart depicting the study selection process.

The total number of articles retrieved from the five databases was 1103 (PubMed n = 207, Web of Science n = 301, Scopus n = 381, Embase n = 68, EBSCOhost n = 146). Additionally, we identified three articles from reference lists. After excluding 487 duplicate articles, the remaining 619 articles underwent an first screening. We excluded 583 articles by evaluating the titles and abstracts. Following a full-text review, 26 articles were excluded. Ultimately, 10 articles met the inclusion criteria. Fig 1 illustrates the detailed screening process.

### 3.2. Characteristics of the included studies

The total number of included studies was 10, involving 270 participants, with 59.63% being male and 40.37% female. The average age of participants was between 11 and 22 years. The exercise intervention varied in duration from 6 to 12 weeks and involved training sessions occurring twice to three times per week. Each training session lasted between 45 and 90 minutes (two studies did not report the duration of each training session). An exhaustive presentation of the detailed characteristics of the included studies can be found in Table 1.

**Table 1:** The characteristics for the studies included.

Study	n	Sex	Age (year)	Height (cm)	Body mass (kg)	Fre	Dur(week)	Time(mins/session)
Bouteraa et al. (2021) [20]	26	F	16.43 ± 0.49	168.0 ± 6.17	56.23 ± 7.69	2	8	45 mins
Chaabene et al. (2021) [8]	23	F	16.85 ± 0.25	164.40 ± 6.47	63.50 ± 4.94	2	8	70 ~ 90 mins
Chaouachi et al. (2014) [21]	28	M	13.50 ± 0.81	159.75 ± 7.89	45.90 ± 8.81	3	8	NR
Guo et al. (2021) [22]	16	M	19.80 ± 1.83	178.45 ± 5.47	68.99 ± 7.89	3	6	60 mins
Guo et al. (2025) [23]	48	M	15.79 ± 0.71	178.44 ± 5.57	68.01 ± 8.09	3	8	60 mins
Lu et al. (2022) [9]	16	M	19.82 ± 1.83	178.44 ± 5.44	69.01 ± 7.09	3	6	60 mins
Makhlouf et al. (2019) [24]	37	M	11.12 ± 0.79	146.25 ± 6.76	36.89 ± 7.84	2	8	NR
Shen (2024) [25]	30	F	15.87 ± 0.78	177.50 ± 3.88	67.73 ± 4.01	3	8	60 mins
Yan et al. (2025) [26]	30	F	22.71 ± 3.82	168.12 ± 5.41	52.08 ± 6.13	3	12	60 mins
Zhou et al. (2022) [27]	16	M	20.50 ± 1.10	177.80 ± 5.10	68.10 ± 7.20	3	6	60 mins

NR = not clearly reported. Fre: Training frequency (session/week). Dur：Duration of time (week).

### 3.3. Results of ROB assessment

**Fig 2:**
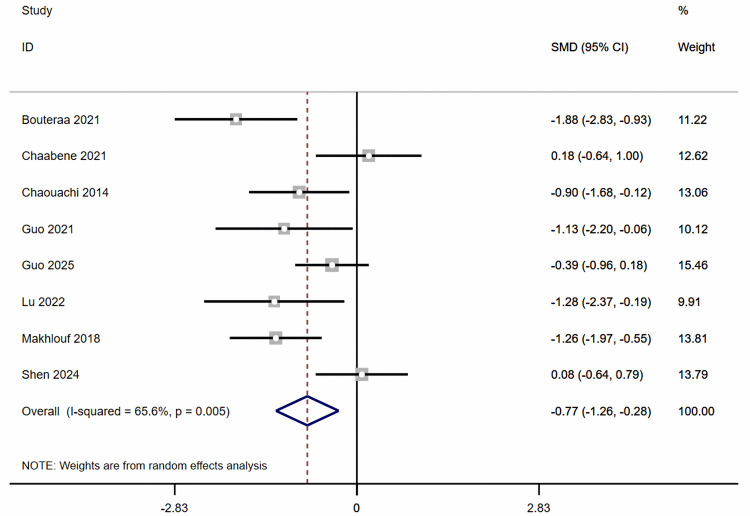
Forest plot showing the effect of combined balance and plyometric training on change-of-direction.

This meta-analysis assessed the methodological quality of the included studies. A single study was identified as being at low risk of bias, six studies had a moderate risk of bias, and three studies had a high risk of bias (other bias risks: fewer than 10 participants in a single group). It is worth noting that, except for Shen (2024) [25], the remaining studies did not clearly report the methods of random allocation and whether allocation concealment was implemented. The results of the bias risk assessment are presented in S2 Figs 1 and 2.

### 3.4. Meta-analysis results

Regarding change-of-direction, the meta-analysis showed (Fig 2) that combined balance and plyometric training significantly improved participants’ change-of-direction ability compared with the control group (k = 8, ES = −0.77, 95% CI: −1.26 to −0.28, *I*^2^ = 65.6%, *P* < 0.05). Subgroup analysis (S1 Table 2) showed no significant differences between subgroups (age, gender, training frequency, duration).

**Fig 3:**
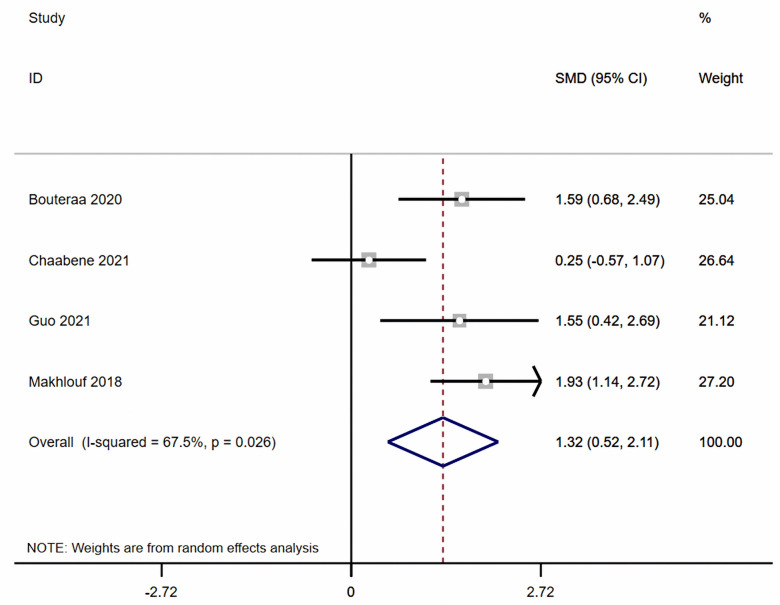
The Forest plots of Y-balance.

**Fig 4:**
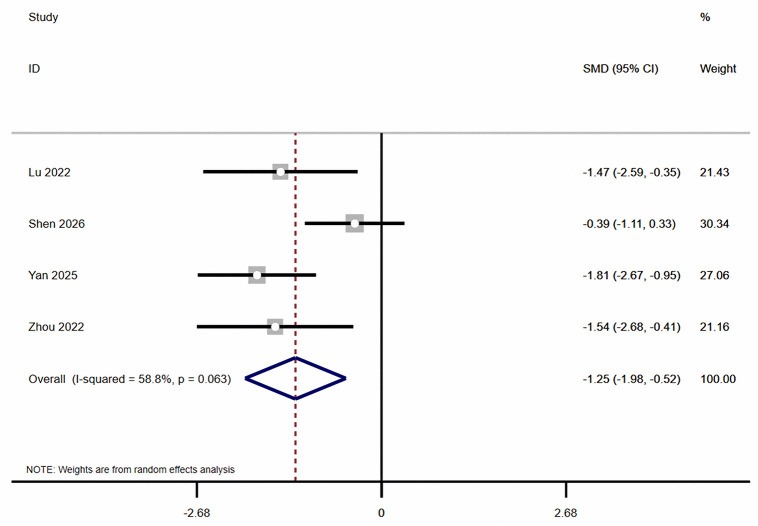
The Forest plots of center of pressure.

**Fig 5:**
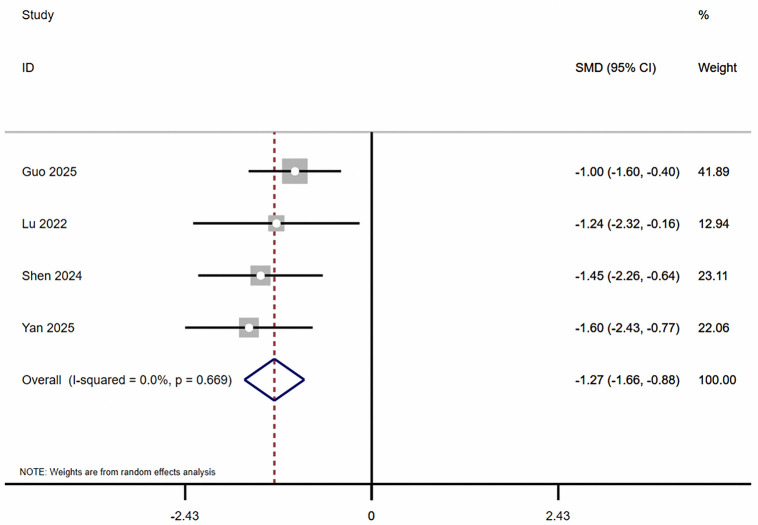
The Forest plots of dynamic postural stability index.

Regarding dynamic balance, this meta-analysis showed (Figs 3–5) that, compared with the control group, combined balance and plyometric training significantly improved participants’ Y-Balance (k = 4, ES = 1.32, 95% CI: 0.52 ~ 2.11, *I*² = 67.5%, *P* < 0.05), Dynamic Postural Stability Index (k = 4, ES = −1.27, 95% CI: −1.66 to −0.88, *I*² = 0.0%, *P* < 0.05), and Center of Pressure (k = 4, ES = −1.25, 95% CI: −1.98 to −0.52, *I*² = 58.8%, *P* < 0.05).

Egger's test results showed that the change-of-direction *P* = 0.49, and the P values of all dynamic balance indicators were greater than 0.4, indicating that there was no publication bias in the change-of-direction and dynamic balance indicators. The sensitivity analysis results showed that after excluding any one study, the direction of the combined effect size did not change, indicating that the results of this meta-analysis were relatively robust.

## 4. Discussion

This meta-analysis included 10 randomized controlled trials involving 270 participants, all of which explored the effects of combined balance and plyometric training on change-of-direction and dynamic balance. The results showed that combined balance and plyometric training significantly improved participants’ change-of-direction and dynamic balance abilities. Subgroup analysis revealed that age, gender, training frequency, and duration exerted no significant influence on the training effect of change-of-direction.

### 4.1. Change-of-direction

The meta-analysis results of this study indicate that combined balance and plyometric training significantly improves participants’ change-of-direction ability (*P* < 0.05), achieving a moderate effect size (ES = −0.77). This finding is largely consistent with previous research conclusions [20,23,25]. Existing studies indicate that change-of-direction (COD) ability is a highly complex motor skill. Its execution relies on multiple movement components, including rapid deceleration, body posture adjustment, and re-acceleration. Consequently, it is influenced not only by lower-body explosive power but also depends on postural control and neuromuscular coordination [1]. For instance, a meta-analysis by Ramirez-Campillo et al. demonstrated that plyometric training significantly enhances basketball players’ change-of-direction speed, primarily through improved eccentric strength and neuromuscular adaptations [13]. Furthermore, Guo et al. (2025) noted that during directional changes, body segments tend to maintain their original motion due to inertia [23]. Consequently, individuals must rely on strong dynamic balance to stabilize their center of gravity and execute efficient directional transitions. These findings collectively support the conclusions of the present study from multiple perspectives.

From the perspective of exercise biomechanics and neuromuscular adaptation mechanisms, the enhancement of change-of-direction ability through combined training likely stems primarily from improvements in stretch-shortening cycle (SSC) efficiency and rate of force development (RFD) [5,6]. During directional changes, athletes must decelerate and generate propulsive force within an extremely brief timeframe, making rate of force development a critical determinant of athletic performance. Plyometric training, through repeated eccentric-concentric muscle contraction stimuli, significantly enhances the muscle's capacity to generate force in short durations while improving the storage and utilization efficiency of elastic potential energy [28]. During the deceleration phase of directional changes, enhanced eccentric strength improves an athlete's capacity to absorb braking forces, enabling faster re-acceleration [29]. Thus, plyometric training provides a crucial strength foundation for improving directional change ability by enhancing RFD and SSC efficiency.

However, the change-of-direction ability does not depend solely on strength levels; it is also significantly influenced by postural control and sensorimotor integration [7,31]. Balance training typically employs unstable support surfaces or disturbance stimuli, compelling the body to continually adjust its postural control strategies [32]. This process strengthens the integration of information between the vestibular, visual, and proprioceptive systems. Enhanced sensorimotor integration enables athletes to rapidly adjust body posture during high-speed movements, maintain center of gravity stability, and improve the precision of turning actions [30]. Research indicates that heightened proprioceptive feedback sensitivity increases joint position and movement awareness, allowing individuals to perceive postural changes more accurately and thereby optimize joint control strategies during directional changes.

Additionally, combined training may further enhance change-of-direction performance through central nervous system adaptation and optimized neuromuscular control. Chaouachi et al. found that during balance and disturbance training, the body gradually develops anticipatory postural adjustments (APA)—preemptively activating stabilizing muscle groups before ground contact or directional shifts to increase joint stiffness and enhance movement stability [21]. This central control strategy significantly improves movement efficiency during deceleration-reacceleration transitions. Concurrently, plyometric training enhances neuromuscular drive in agonist muscles and improves intermuscular and intramuscular coordination, thereby increasing the nervous system's capacity to regulate rapid movement patterns [33]. Thus, combining balance and plyometric training simultaneously promotes agility development by enhancing both force production capacity and neuromuscular control capabilities.

It is noteworthy that the results of this study indicate a certain degree of heterogeneity in the analysis of change-of-direction ability (*I*² = 65.6%). Possible reasons for this phenomenon include differences in the testing tools used across studies, such as the Modified T-Test, Illinois Agility Test, and Sport-Specific Agility Test. These tests involve varying turning angles, distances, and movement patterns, which may influence the assessment of training intervention effects. Furthermore, variations in training duration, frequency, and intensity across studies may also influence outcomes. Therefore, future research should standardize assessment methods for change-of-direction ability and establish standardized training intervention protocols to enhance the comparability of research findings.

### 4.2. Dynamic balance

Regarding dynamic balance, the findings of this study indicate that combined balance and plyometric training significantly improved the Y-balance test, dynamic postural stability index, and pressure center metrics (*P* < 0.05), demonstrating substantial effect sizes. These results are largely consistent with previous research conclusions [9,26]. Asadi et al. found that a 6-week plyometric training program significantly improved athletes’ performance on the Star Test, primarily due to enhanced neuromuscular control [30]. Concurrently, Zhou et al. indicated that balance training enhances ankle joint stability and optimizes the efficiency of the postural control system in utilizing diverse sensory information by strengthening neuromuscular control and proprioceptive feedback [27]. These findings collectively support the promotional effect of combined training on dynamic balance capacity [34].

From the perspective of sensory-motor control mechanisms, improvements in dynamic balance may primarily stem from heightened sensitivity within the proprioceptive system [35,36]. During balance training, the body must continually respond to disturbances from unstable environments. This repetitive stimulation activates mechanoreceptors around joints and enhances the sensitivity of sensory afferent signals [37]. As training progresses, the central nervous system becomes more efficient at integrating information from visual, vestibular, and proprioceptive systems, thereby developing more stable and precise postural control strategies. Consequently, the enhancement of proprioceptive feedback capacity is considered a crucial physiological foundation for improving dynamic balance ability.

Secondly, peripheral neuromuscular adaptation may also play a crucial role in enhancing dynamic balance. Plyometric training subjects the tendon-muscle complex to rapid changes in length and tension under fast eccentric loading conditions. This mechanical stimulus may promote functional adaptation of muscle spindles and the Golgi tendon organ [38,39]. Research indicates that changes in tendon organ sensitivity can enhance the muscle spindle system's responsiveness to muscle length variations, thereby improving muscle perception of joint movement [40]. This heightened sensory feedback capability enables individuals to more accurately regulate joint position and movement trajectories, ultimately enhancing dynamic postural control.

Additionally, adaptive changes in the central nervous system may also serve as a key mechanism through which combined training improves dynamic balance [41]. Horak (2006) demonstrated that balance training and motor skill training enhance the body's ability to respond to disturbance stimuli by promoting feedforward postural adjustment mechanisms and strengthening the preactivation capacity of stabilizing muscle groups [42]. This feedforward control strategy enables the body to activate muscles in advance of external disturbances, thereby reducing postural adjustment latency and enhancing stability. Concurrently, plyometric training improves the nervous system's efficiency in controlling rapid movements, leading to significant improvements in muscle reaction speed and coordination. Thus, the combination of these two training modalities fosters synergistic adaptation across sensory input, neural integration, and motor output, resulting in a marked enhancement of dynamic balance ability [43].

Notably, among all indicators in this study, the dynamic postural stability index exhibited the lowest heterogeneity (*I*² = 0%), indicating high stability and consistency in evaluating training intervention effects. In contrast, the Y-balance test and center of pressure metrics exhibited higher heterogeneity, potentially attributable to variations in testing postures, measurement devices, and data processing methods across different studies. Therefore, future research should prioritize standardized testing methods when evaluating dynamic balance capabilities to enhance the reliability and comparability of findings.

### 4.3. Practical implications

The findings of this study indicate that combining balance and plyometric training can significantly enhance athletes’ change-of-direction ability and dynamic balance within a relatively short training cycle. From a training perspective, this approach simultaneously stimulates neuromuscular strength output and postural control, creating a more comprehensive pathway for athletic performance enhancement. Therefore, when designing physical training programs, coaches can effectively combine unstable support training, single-leg balance exercises, and multi-directional jumping drills to improve athletes’ body control in high-speed movement environments. Furthermore, for youth athletes or sports requiring frequent directional changes (such as basketball, soccer, and badminton), combined training protocols may enhance performance while reducing lower-limb injury risks. However, future research should further explore the effects of varying training durations, intensities, and sport-specific adaptations through long-term intervention studies.

### 4.4. Limitations

Although this meta-analysis comprehensively evaluated the effects of combined balance and plyometric training on change-of-direction and dynamic balance, there are still certain limitations. First, due to inconsistencies in measurement tools and training programs, this meta-analysis has heterogeneity. Second, owing to the paucity of extant original studies, the analysis of subgroups was constrained to the change-of-direction indicator, while the remaining indicators were not analyzed in subgroups and regression analysis. Finally, among the 10 studies, only one was rated as having a low risk of bias in the Cochrane bias risk assessment. A majority of studies did not adequately document their methods of randomization, whether allocation concealment was implemented, or whether outcome measures were assessed using a blinded method. Therefore, future studies need to further improve the methodological quality of their research.

## 5. Conclusions

This study found through meta-analysis that combined balance and plyometric training significantly enhances individuals’ change-of-direction ability and dynamic balance. At the mechanistic level, combined training may optimize the neuromuscular system's coordinated regulation of rapid movement and postural stability by simultaneously promoting lower-limb strength development rate, eccentric-concentric cycle efficiency, and improvements in proprioceptive feedback and postural control. This provides new evidence supporting the training adaptation mechanisms underlying change-of-direction ability and dynamic balance. From a training practice perspective, combining balance and plyometric training can enhance dynamic postural control while improving athletic performance. Therefore, it serves as a crucial physical training method for sports requiring frequent directional changes and rapid stability control, such as basketball, soccer, and badminton. However, given the remaining research heterogeneity in certain indicators, future studies should further clarify the optimal training duration and load for combined training through larger sample sizes and standardized testing methods.

## Supporting information

S1 Table Detailed search strategy. (DOCX)

S2 Fig Methodological quality assessment. (DOCX)

S3 Table PRISMA 2020 checklist. (PDF)
