# Supplementary material for: The effects of combined balance and plyometric training on change-of-direction and dynamic balance: A meta-analysis
Source: PLoS One. 2026 Mar 31;21(3):e0346232. doi: 10.1371/journal.pone.0346232 (PMC13037961; doi:10.1371/journal.pone.0346232)
Supplement: S1 Table — (DOCX) [file pone.0346232.s001.docx]

**The effects of combined balance and plyometric training on change-of-direction and dynamic balance: a meta-analysis**

Guang Feng^1^, Ruobing Chen^1^, Chonghui Wu^2^, Yongfeng Liu^1*^

^1^ School of Sports Training, Chengdu Sport University, Shanghai, China

^2^ School of Foreign Languages, Xihua University, Chengdu, China

^*^Corresponding Author:

E-mail: [lyf197707@163.com](mailto:lyf197707@163.com) (YFL)

**Supplementary Table 1 The Search strategies for different data**

| **Data** | **Query** | **Results** |
| --- | --- | --- |
| PubMed | (plyometric[Title/Abstract]) OR (force-velocity[Title/Abstract]) OR (stretch–shortening cycle[Title/Abstract]) AND （balance[Title/Abstract]) OR （instability training[Title/Abstract]) OR （posture control[Title/Abstract]) | 207 |
| Web of Science | ((TS=Plyometric OR force-velocity OR stretch–shortening cycle)) AND ((TS=balance OR instability training OR posture control)) | 301 |
| Scopus | ("Plyometric" OR "force-velocity" OR "stretch–shortening cycle") in Title Abstract Keyword AND ("balance" OR "instability training" OR "posture control") in Title Abstract Keyword | 381 |
| Embase | ('Plyometric':ab,ti OR 'force-velocity':ab,ti OR 'stretch–shortening cycle':ab,ti) AND ('balance':ab,ti OR 'instability training':ab,ti OR 'posture control':ab,ti) AND (combine*':ab,ti OR 'combination':ab,ti) | 68 |
| EBSCOhost | (AB Plyometric OR AB force-velocity OR AB stretch–shortening cycle) AND (AB balance OR AB instability training OR AB posture control) AND (AB combine* OR AB combination) | 146 |

**Supplementary Table 2 The Subgroup analyses of** **change-of-direction**

| **Subgroup** | **K** | **N** | **Hedges' *g*** **(95%CI)** | ***Pd*** | ***I*^2^** | ***Pb*** |
| --- | --- | --- | --- | --- | --- | --- |
| **Sex** |  |  |  |  |  | 0.401 |
| Male | 5 | 145 | -0.88 (-1.26, -0.50) | 0.332 | 14.5% |  |
| Females | 3 | 79 | -0.51 (-1.72, 0.70) | 0.002 | 84.5% |  |
| **Age** |  |  |  |  |  | 0.507 |
| ≤ 15.87 year | 4 | 143 | -0.60 (-1.16, -0.05) | 0.051 | 61.5% |  |
| ＞15.87 year | 4 | 81 | -1.00 (-1.95, -0.05) | 0.01 | 73.7% |  |
| **Training frequency** |  |  |  |  |  | 0.605 |
| ≤ 2 per week | 3 | 86 | -0.97 (-2.12, 0.18) | 0.003 | 82.9% |  |
| ＞2 per week | 5 | 138 | -0.61 (-1.09, -0.14) | 0.142 | 41.9% |  |
| **Weeks of duration** |  |  |  |  |  | 0.430 |
| ≤ 6 weeks | 6 | 32 | -1.21 (-1.97, -0.44) | 0.843 | 0.0% |  |
| ＞6 weeks | 2 | 192 | -0.66 (-1.24, -0.08) | 0.003 | 72.6% |  |

Notes: *p* value, statistically significant p values for pooled results; *I* ^2^, quantitative indicators of heterogeneity; Hedge's *g*, the effect size indicators used in the pooled;

Abbreviations: 95%CI = 95% confidence interval; K = the total number of effects included in the pooled effect size; N = sample size;
