# Supplementary material for: The effects of combined balance and plyometric training on change-of-direction and dynamic balance: A meta-analysis
Source: PLoS One. 2026 Mar 31;21(3):e0346232. doi: 10.1371/journal.pone.0346232 (PMC13037961; doi:10.1371/journal.pone.0346232)
Supplement: S2 Fig — (DOCX) [file pone.0346232.s002.docx]

**The effects of combined balance and plyometric training on change-of-direction and dynamic balance: a meta-analysis**

Guang Feng^1^, Ruobing Chen^1^, Chonghui Wu^2^, Yongfeng Liu^1*^

^1^ School of Sports Training, Chengdu Sport University, Shanghai, China

^2^ School of Foreign Languages, Xihua University, Chengdu, China

^*^Corresponding Author:

E-mail: [lyf197707@163.com](mailto:lyf197707@163.com) (YFL)


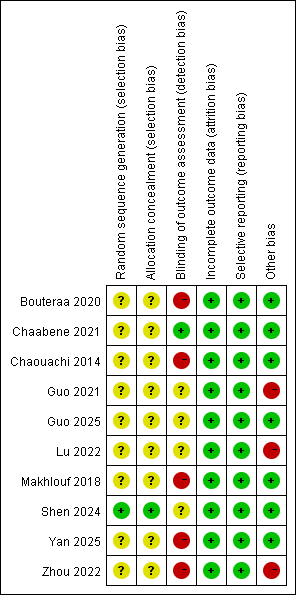


**Supplementary Fig 1. Assessment of methodological quality of included studies**


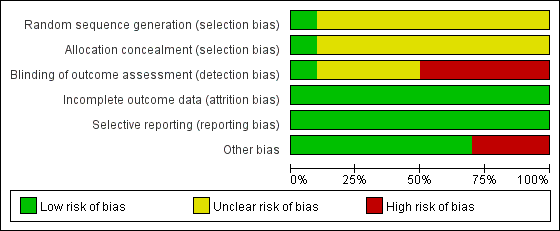


**Fig 2. Percentage of studies included in the methodological quality assessment**
